# Neopterin as a Tool for Primate Ecoimmunology: Current Knowledge, Practical Application, and New Directions From Captivity to the Wild

**DOI:** 10.1002/ajp.70081

**Published:** 2025-10-15

**Authors:** Verena Behringer, Caroline Deimel

**Affiliations:** ^1^ Endocrinology Laboratory, German Primate Center, Leibniz Institute for Primate Research Göttingen Germany; ^2^ Department of Behavioral and Cognitive Biology Faculty of Life Sciences University of Vienna Vienna Austria

**Keywords:** cell‐mediated immunity, immunoecology, macrophage activation, sample handling, Th1 response, urine biomarker

## Abstract

Neopterin is a well‐established biomarker of interferon‐gamma‐mediated macrophage activation that indicates cell‐mediated immune system responses in humans. Because it is readily quantifiable in urine, it is increasingly used in nonhuman primates to study cell‐mediated immune functioning in relation to infectious diseases, but also environmental and individual factors, in both captive and wild primates. This review synthesizes our current knowledge on these topics with a focus on nonhuman primates. We cover the influence of various methodological factors during sampling and analysis on the reliability of neopterin measurements and give practical advice on how these factors can be mitigated. Furthermore, we address the advantages and disadvantages of different biological matrices in which neopterin can be measured and propose best practice guidelines for handling and storage of samples that consider challenges encountered during fieldwork. We conclude this review with an outlook on topics within primatology where neopterin, as a marker of cell‐mediated immune functioning, could become a valuable tool to answer applied questions and test evolutionary hypotheses about immune functioning in primates.

## Summary

- Neopterin is a sensitive and specific biomarker of cell‐mediated immune activation, responsive to intracellular pathogens and chronic inflammation, and affected by environmental, life‐history, and sex‐contextual factors.
- It can be reliably quantified in urine, allowing frequent sampling in captive and habituated wild primates. This review offers best‐practice guidelines and practical advice on sample collection, storage, and analysis.
- Neopterin provides a valuable tool for primate ecoimmunology, for investigations of immune responses and trade‐offs in ecologically relevant contexts and can help to evaluate conservation and welfare interventions.

## Graphical abstract

Neopterin is a sensitive and specific biomarker of intracellular pathogen infection and chronic inflammation, and affected by environmental, life‐history, and sex‐contextual factors. This review offers best‐practice guidelines and practical advice on sample collection, storage, and analysis in primates when used for ecoimmunological conservation and welfare questions.

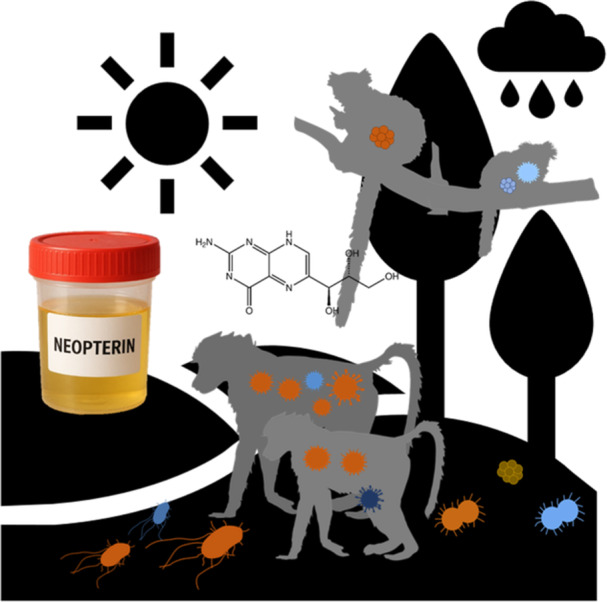

## 1 Introduction

Trade‐offs between immune functioning and other life‐history events are central to many hypotheses in evolutionary ecology (e.g., Demas and Nelson 2012; McDade et al. 2016; Sheldon and Verhulst 1996), and ecoimmunology has become increasingly relevant for conservation biology and animal welfare science (Düpjan and Dawkins 2022; Ohmer et al. 2021; Schulenburg et al. 2009). For example, investment in immune functioning may be reduced during energetically demanding reproductive periods or increased during infection at the expense of somatic growth (Audzijonyte and Richards 2018; Edward and Chapman 2011). Understanding how these trade‐offs between growth, reproduction, and maintenance are negotiated within individuals who are embedded in complex social networks, while also being shaped by past and current environmental conditions is important for understanding variation in primate health, disease susceptibility, and adaptive capacity (Behringer et al. 2021; Kinnally et al. 2019; MacIntosh et al. 2012; Thompson González et al. 2025). However, ecoimmunological frameworks have so far largely overlooked the variety and specificity of immune system functioning, potentially limiting their explanatory power and our understanding of these complex interactions in primates (Dibakou et al. 2020). One major constraint is that methods to study immune system functioning in wild primates are limited, as blood sampling is usually not feasible, and only a limited number of immune‐biomarkers can be reliably measured in urine or fecal samples (Higham 2016; Urlacher et al. 2022).

Neopterin is a biomarker that reflects the activation of cell‐mediated immune responses in primates, including humans (e.g., Fuchs et al. 1992; Hamerlinck 1999; Hoffmann and Schobersberger 2004). Its use is well‐established in human clinical research and in recent years it is increasingly recognized as a valuable tool in primatology because it can be reliably quantified in urine samples. Here, we provide a practical guide for interested primatologists that synthesizes our current knowledge on neopterin and its application in nonhuman primate research. In this review, we explain where neopterin is produced, what its presence can and cannot tell us about immune activation and clarify its specificity for cell‐mediated immune responses, particularly in reaction to intracellular pathogens. We review empirical findings on neopterin patterns in relation to infections, general health, inflammation, and aging in primates, including human studies when necessary. We also summarize findings of neopterin patterns in relation to environmental and individual factors. Finally, we provide practical recommendations for sample collection, handling, and neopterin analyses. By consolidating this information, we aim to support primatologists in effectively integrating neopterin into their research and to encourage new studies and collaborations addressing the complex questions of primate health, disease ecology, and OneHealth perspectives.

## 2 Understanding Neopterin: What Does It Measure?

The immune system is a complex system that protects organisms from pathogens and malignant cells by recognizing and responding to these threats while distinguishing them from the body's own healthy tissues. A detailed overview of the immune system's functioning is beyond the scope of this review (for comprehensive overviews, see Actor 2014; Clark 2008; Janeway 2005). However, to contextualize the specificity, utility, and limitations of neopterin as a biomarker of cell‐mediated immune activation in primates, it is essential to introduce key terminology and background information. Therefore, we provide a brief overview of the different conceptual ways the immune system is organized and highlight the immune cells and signaling pathways that are directly involved in neopterin production.

### 2.1 Immunological Basics Relevant to Neopterin

It is common knowledge that the vertebrate immune system is organized into two lines of defense: the innate and the adaptive immune system. The innate immune system constitutes the first line of defense and is characterized by rapid, nonspecific responses that do not vary with pathogen type (Knapp and Torosin 2019). These responses are genetically encoded based on pathogen components, are not shaped by prior exposure, and are activated by conserved pathogen‐associated molecular patterns. In addition to providing immediate protection, the innate immune system also facilitates the activation of the second line of defense, the adaptive immune system (Abbas et al. 2018; Murphy and Weaver 2018). The adaptive immune system is slower to respond but shows high specificity, as it can distinguish self from nonself and generate targeted responses to specific pathogens. A key feature of adaptive immunity is immunological memory, which allows a more rapid and robust response upon subsequent exposure to the same pathogen, a principle that underlies vaccine efficacy (Abbas et al. 2018; Janeway 2005). Each system comprises two principal types of immune responses: cell‐mediated and humoral immunity (Castelo‐Branco and Soveral 2014; Delves and Roitt 2000; Nicholson 2016). Cell‐mediated immunity relies on the activation of immune cells, such as T lymphocytes and macrophages, whereas humoral immunity is mediated by macromolecules, notably antibodies, present in extracellular fluids (Chaplin 2010; Nicholson 2016).

### 2.2 Neopterin as an Indicator of Cell‐Mediated Immune Activation

When assessing immune system functioning through the use of biomarkers, it is essential to understand which specific component of the immune response a reflected by a given marker. Neopterin is associated with cell‐mediated immune responses that primarily target intracellular pathogens like viruses, but also certain bacteria and protozoa. These responses involve T helper type 1 (Th1) cells and macrophages (Berger 2000; O'Garra and Arai 2000). T helper (Th) cells play a critical role in adaptive immunity by orchestrating immune responses through their cytokine secretion profiles. Th1 cells are primarily involved in cell‐mediated immunity, promoting responses effective against intracellular pathogens such as viruses and certain bacteria. They secrete cytokines including interferon‐gamma (IFN‐γ), interleukin‐2 (IL‐2), and tumor necrosis factor‐beta (TNF‐β), which activate macrophages and cytotoxic T cells (Abbas et al. 2018; Chaplin 2010). In contrast, Th2 cells support humoral immunity by facilitating B cell activation and antibody production through the secretion of cytokines such as interleukin‐4 (IL‐4), IL‐5, and IL‐13. This response is particularly effective against extracellular pathogens and parasites (Berger 2000; Muraille and Leo 1998; O'Garra and Arai 2000). The balance between Th1 and Th2 responses is crucial for an appropriate immune reaction and is often explored in disease pathogenesis and immunoregulation (Romagnani 2006). Understanding these distinct pathways is essential for interpreting neopterin as a biomarker for immune system activation. Neopterin primarily reflects Th1‐driven cellular immune activation (Fuchs et al. 1989; Huber et al. 1984).

Neopterin is produced and released by macrophages, dendritic cells, and monocytes upon activation by IFN‐γ, a cytokine secreted predominantly by Th1 cells of the adaptive immune system and by natural killer (NK) cells of the innate immune system (Fuchs et al. 1994; Murr et al. 2002). IFN‐γ is released in response to viral infections, intracellular bacteria, and antigens (Schoenborn and Wilson 2007). Therefore, increased neopterin levels serve as a reliable indicator of Th1‐mediated immune activation and reflect the cellular immune response to intracellular immune challenges (Eisenhut 2013; Fuchs et al. 1988, 1989; Gieseg et al. 2018; Huber et al. 1983, 1984; Wachter et al. 1992). It is important to acknowledge that neopterin reflects this specific facet of the multifaceted immune response and does not represent the entirety of immunological processes.

## 3 Neopterin: A Unique Immune Marker in Primates

Neopterin (2‐amino‐4‐hydroxy‐6‐(d‐erythro‐1′,2′,3′‐tryhydroxypropyl)‐pteridine) is a member of the pteridine class of heterocyclic compounds. It is not a hormone, as it lacks regulatory signaling functions typically associated with hormone activity. Instead, its production serves a protective role in macrophages by counteracting oxidative stress and preventing cell death during inflammatory processes (Heneberk et al. 2023). By interfering with cellular redox systems, it also mediates host–defense reactions (Hoffmann et al. 2003; Hoffmann and Schobersberger 2004).

Given its role in neutralizing free radicals, neopterin production is closely linked with reactive oxygen species (ROS) formation, particularly in IFN‐γ‐stimulated macrophages (Murr et al. 2002; Sucher et al. 2010). This association suggests that neopterin may serve as a biomarker of both oxidative stress and ROS activity (Gieseg et al. 2018; Murr et al. 1999; Sucher et al. 2010).

### 3.1 Biosynthesis of Neopterin

**Figure 1:**
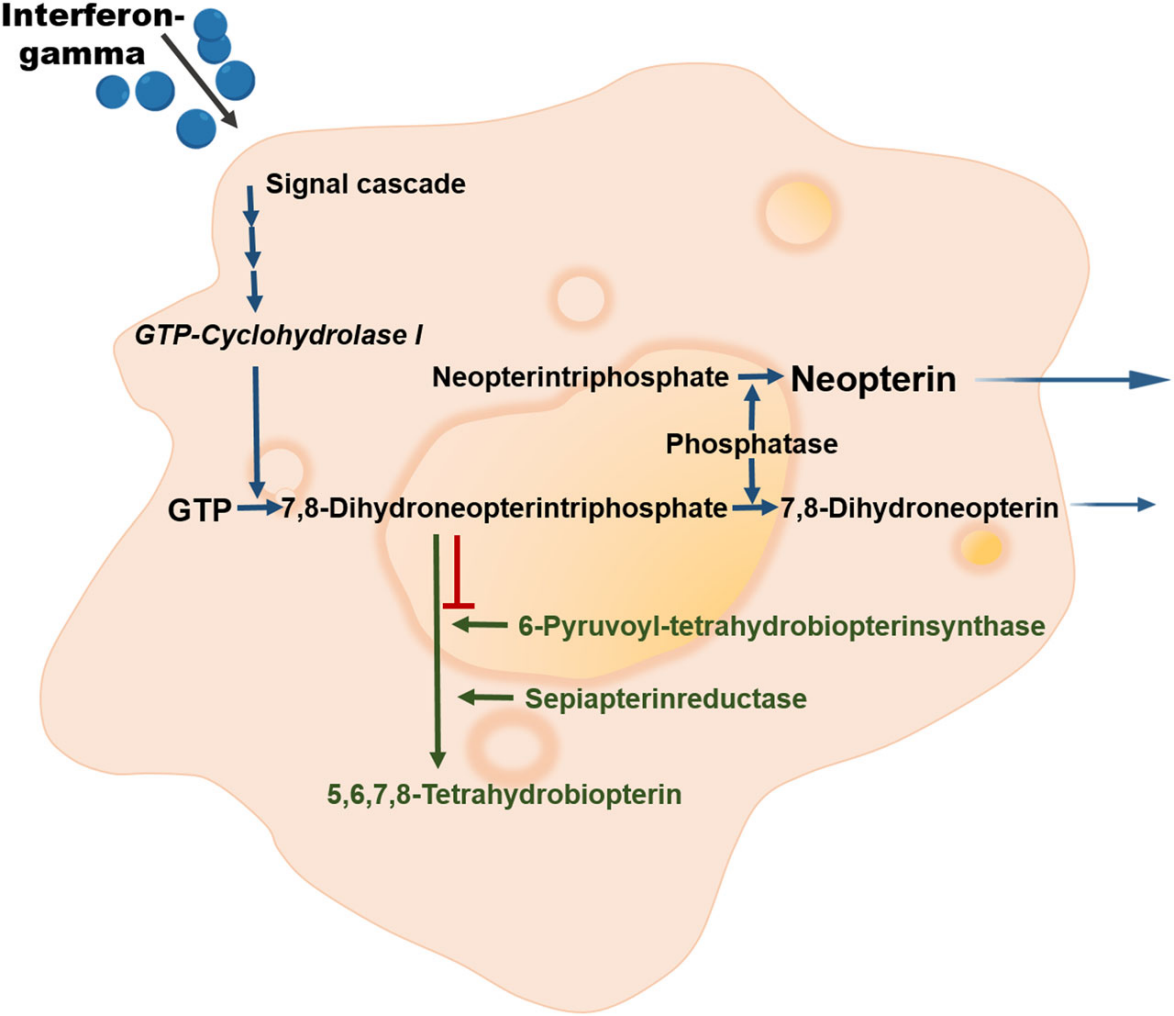
Biosynthesis of neopterin derivatives in human monocytes/macrophages. Interferon‐gamma (blue) induces guanosine triphosphate (GTP) cyclohydrolase, which catalyzes the conversion of GTP to 7,8‐dihydroneopterin. Due to a constitutive deficiency (shown in red) of 6‐pyruvoyl tetrahydrobiopterin synthase in human macrophages, neopterin derivatives accumulate at the expense of biopterin derivatives. Modified after Murr et al. (2002).

Neopterin synthesis is initiated when IFN‐γ‐stimulated macrophages convert guanosine triphosphate (GTP) into 7,8‐dihydroneopterin, a reaction catalyzed by cyclohydrolase I (Figure 1). In primates, a relative deficiency in 6‐pyruvoyl‐tetrahydropterin synthase leads to an accumulation of 7,8‐dihydroneopterin within the cytoplasm of monocytes and macrophages, where it reacts with oxidants to produce neopterin (Gieseg et al. 2018). Therefore, primate macrophages and monocytes produce neopterin in particularly high amounts compared to other neopterin derivatives. Studies in humans have shown that the diagnostic information obtained from measuring these other derivatives is comparable to that of neopterin (Fuchs et al. 1989; Wachter et al. 1992).

### 3.2 Applications in Nonhuman Primate Studies and Expansion Across Taxa

To date, neopterin has been quantified in several species of haplorrhine primates. A recent publication has also measured urinary neopterin in two strepsirrhini species (Guevara et al. 2025). We have summarized these studies in Table 1. To expand our understanding of neopterin's presence and quantifiability across a broader phylogenetic spectrum, we conducted pilot assay runs on several primate species available to us, including several strepsirrhines. This study underscores the growing interest and feasibility of assessing neopterin across a more diverse range of primates.

**Table 1:** Primate species in which neopterin has been quantified, including taxonomic classification, study environment, biological matrix, and references.

Family	Genus	Species name	Scientific name	Environment	Matrix	Reference
Atelidae	*Alouatta*	Colombian red howler	*Alouatta seniculus*	Wild	Urine	Sacco et al. (2020)
Callitrichidae	*Leontocebus*	Weddell's saddle‐back tamarin	*Leontocebus weddelli*	Wild	Urine	Sacco et al. (2020)
Callitrichidae	*Leontopithecus*	Golden lion tamarin	*Leontopithecus rosalia*	Zoo	Urine	Sacco et al. (2020)
Callitrichidae	*Saguinus*	Emperor tamarin	*Saguinus imperator*	Wild	Urine	Sacco et al. (2020)
Cebidae	*Cebus*	White‐faced capuchin	*Cebus imitator*	Wild	Urine	Lucore et al. (2022)
Cebidae	*Cebus*	White‐faced capuchin	*Cebus imitator*	Wild	Urine	Lucore et al. (2024)
Cebidae	*Sapajus*	Tufted capuchin	*Sabajus apella*	Laboratory	Blood	Lahoz et al. (2009)
Cebidae	*Sapajus*	Tufted capuchin	*Sabajus apella*	Zoo	Urine	Lucore et al. (2022)
Cercopithecidae	*Cercopithecus*	Blue monkey	*Cercopithecus mitis*	Free‐ranging	Urine	Thompson González et al. (2025)
Cercopithecidae	*Macaca*	Barbary macaques	*Macaca sylvanus*	Free‐ranging	Urine	N. Müller et al. (2017)
Cercopithecidae	*Macaca*	Long‐tailed macaque	*Macaca fascicularis*	Laboratory	Feces, urine, blood	Higham et al. (2015)
Cercopithecidae	*Macaca*	Long‐tailed macaque	*Macaca fascicularis*	Laboratory	Urine	Stahl‐Hennig et al. (2002)
Cercopithecidae	*Macaca*	Rhesus macaque	*Macaca mulatta*	Laboratory	Urine	Heistermann and Higham (2015)
Cercopithecidae	*Macaca*	Rhesus macaque	*Macaca mulatta*	Laboratory	Urine	Fendrich et al. (1989)
Cercopithecidae	*Macaca*	Rhesus macaque	*Macaca mulatta*	Free‐ranging	Urine	Cooper et al. (2022)
Cercopithecidae	*Macaca*	Rhesus macaque	*Macaca mulatta*	Laboratory	Feces, urine, blood	Higham et al. (2015)
Cercopithecidae	*Macaca*	Rhesus macaque	*Macaca mulatta*	Laboratory	Urine	Stahl‐Hennig et al. (2002)
Cercopithecidae	*Macaca*	Rhesus macaque	*Macaca mulatta*	Free‐ranging	Urine	Cooper et al. (2025)
Cercopithecidae	*Mandrillus*	Mandrill	*Mandrillus sphinx*	Wild	Blood	Dibakou et al. (2020)
Cercopithecidae	*Mandrillus*	Mandrill	*Mandrillus sphinx*	Free‐ranging	Feces	Dibakou et al. (2019)
Cercopithecidae	*Papio*	Chacma baboon	*Papio ursinus*	Laboratory	Blood	Strohmaier et al. (1991)
Cercopithecidae	*Papio*	Chacma baboon	*Papio ursinus*	Laboratory	Blood	Redl et al. (2000)
Cercopithecidae	*Theropithecus*	Gelada	*Theropithecus gelada*	Wild	Urine	Schneider‐Crease et al. (2022)
Hominidae	*Pan*	Bonobo	*Pan paniscus*	Wild	Urine	Behringer et al. (2017)
Hominidae	*Pan*	Bonobo	*Pan paniscus*	Wild, zoo	Urine	Behringer et al. (2024)
Hominidae	*Pan*	Bonobo	*Pan paniscus*	Wild	Urine	Kreyer et al. (2023)
Hominidae	*Pan*	Bonobo	*Pan paniscus*	Wild, zoo	Urine	Behringer et al. (2021)
Hominidae	*Pan*	Chimpanzee	*Pan troglodytes*	Wild	Urine	Thompson González et al. (2020)
Hominidae	*Pan*	Chimpanzee	*Pan troglodytes*	Wild	Urine	Negrey et al. (2021)
Hominidae	*Pan*	Chimpanzee	*Pan troglodytes*	Wild	Urine	Wu et al. (2018)
Hominidae	*Pan*	Chimpanzee	*Pan troglodytes*	Wild, zoo	Urine	Behringer et al. (2019)
Hominidae	*Pan*	Chimpanzee	*Pan troglodytes*	Wild	Urine	Löhrich et al. (2018)
Indriidae	*Propithecus*	Coquerel's sifaka	*Propithecus coquereli*	Captive	Urine	Guevara et al. (2025)
Lemuridae	*Lemur*	Ring‐tailed lemur	*Lemur catta*	Captive	Urine	Guevara et al. (2025)
Pitheciidae	*Pithecia*	White‐faced saki	*Pithecia pithecia*	Zoo	Urine	Sacco et al. (2020)
Pitheciidae	*Plecturocebus*	Toppin's titi monkey	*Plecturocebus toppini*	Wild	Urine	Sacco et al. (2020)

#### 3.2.1 Ethics Statement

All procedures were entirely noninvasive and complied with the ASAB/ABS Guidelines for the Use of Animals in Research (https://www.asab.org/ethics). The research adhered to the American Society of Primatologists (ASP) Principles for the Ethical Treatment of Nonhuman Primates and followed the ASP Code of Best Practices for Field Primatology.

#### 3.2.2 Assay Methods

For these additional analyses, urine samples were thawed, vortexed, and centrifuged prior to processing. To determine the appropriate dilution factors for the neopterin assay, we initially selected a random subset of samples from 13 primate species (see Table 2) and performed serial dilutions (1:10, 1:50, 1:200) using the assay buffer provided by the manufacturer. Based on initial results of this first assay plate and specific gravity (SG) measurements, optimal species‐specific dilutions were established. SG was measured in all samples using a handheld refractometer to account for variation in both urine volume and concentration (Miller et al. 2004).

**Table 2:** Urinary neopterin levels (ng/mL corrected for specific gravity (SG)) in strepsirrhines and other primates, including sample size (*N*), mean, median, standard deviation (SD), standard error of the mean (SEM), and range (Min–Max).

Suborder	Family	Species	Scientific name	Environment	*N*	Mean (ng/mL corr. SG)	Median (ng/mL corr. SG)	SD	SEM	Min.	Max.
Strepsirrhini	Lemuridae	Black‐and‐white ruffed lemur	*Varecia variegata*	Captive	4	66.26	39.52	34.69	39.52	18.0	168.0
Strepsirrhini	Lemuridae	Crowned lemur	*Eulemur coronatus*	Captive	4	11.24	9.98	4.83	2.42	6.9	18.1
Strepsirrhini	Lemuridae	Red‐bellied lemur	*Eulemur rubriventer*	Captive	4	11.02	11.20	7.36	3.68	3.2	18.5
Strepsirrhini	Lemuridae	Red‐fronted lemur	*Eulemur rufifrons*	Captive	4	23.70	16.64	15.04	7.52	12.4	45.1
Haplorhini	Callitrichidae	Common marmoset	*Callithrix jacchus*	Captive	4	846.77	768.21	171.84	85.92	746.7	1104.0
Haplorhini	Cercopithecidae	Assamese macaque	*Macaca assamensis*	Wild	3	429.32	444.44	158.78	91.67	263.5	580.0
Haplorhini	Cercopithecidae	Moor macaque	*Macaca maura*	Wild	4	205.58	208.48	41.11	20.56	152.7	252.6
Haplorhini	Cercopithecidae	Celebes crested macaque	*Macaca nigra*	Wild	4	144.62	155.91	50.14	25.07	80.0	186.7
Haplorhini	Cercopithecidae	Northern plains gray langur	*Semnopithecus entellus*	Captive	4	64.90	56.19	36.75	18.38	34.3	112.9
Haplorhini	Cercopithecidae	Olive baboon	*Papio h. anubis*	Captive	4	341.62	315.56	244.61	122.31	120	615.4
Haplorhini	Hominidae	Western lowland gorilla	*Gorilla gorilla gorilla*	Captive	4	117.81	108.89	48.66	24.33	70.6	182.9
Haplorhini	Hominidae	Sumatra orangutan	*Pongo abelii*	Captive	4	184.70	306.54	64.32	32.16	194.3	331.4

*Note:* Animals are grouped by suborder, family, and species, with sampling environment indicated.

In a second assay plate, the following dilutions were applied: ape, baboon, and langur samples were diluted 1:50; lemur samples 1:10; and marmoset samples 1:400. Due to particularly high variation in SG among macaques, their samples were diluted at 1:50, 1:200, or 1:400, depending on individual SG values.

Neopterin levels were determined using a commercial competitive enzyme‐linked immunosorbent assay (Neopterin ELISA, Ref. RE59321, IBL International GmbH, Hamburg, Germany), originally developed for use with human serum, plasma, and urine, and previously validated for application in nonhuman primates. All assay steps followed the manufacturer's protocol. Briefly, 20 μL of diluted urine, 100 μL of enzyme conjugate, and 50 μL of neopterin antiserum were added to each well. Plates were incubated in the dark for 90 min, followed by four washing steps using the provided buffer. Subsequently, 150 μL of tetramethylbenzidine substrate solution was added, and the reaction was stopped after 10 min with 150 μL of stop solution. Absorbance was measured at 450 nm using a microplate reader. All samples, standards, and controls were measured in duplicate. Inter‐assay coefficients of variance (CV) of quality controls of high and low concentrations were 2.2% and 5.2% (*N* = 2) and intra‐assay CV was 2.6% (*N* = 37).

#### 3.2.3 Neopterin in Strepsirrhines and Other Primates

The urinary neopterin results of our pilot study are shown in Table 2. We were able to measure neopterin in all additional species, also in strepsirrhines. However, the urinary neopterin levels measured in lemurs were low compared to other species, which may reflect a species‐specific metabolic difference: strepsirrhines may lack the relative deficiency in 6‐pyruvoyl‐tetrahydropterin synthase observed in other primates that leads to neopterin accumulation (Gieseg et al. 2018; Murr et al. 1999). Furthermore, it remains unclear whether the samples came from individuals experiencing acute infections, so we cannot determine if neopterin reflects immune activation in response to intracellular pathogens in these species, as it does in haplorrhine primates. Researchers interested in using urinary neopterin as a biomarker in nonhuman primates should validate the assay for each species before broader application. While we emphasize the importance of confirming that neopterin reflects cell‐mediated immune responses, it is equally critical to establish that the immunoassay specifically and accurately measures the neopterin molecule in the species under study. Assays developed for humans cannot be assumed to perform identically in other taxa due to potential structural differences in the molecule or its epitopes, which can affect antibody binding and, consequently, assay accuracy. Without such validation, measured neopterin concentrations may be misleading, as low readings could reflect poor assay performance rather than true biological differences. Therefore, careful biochemical and immunological validation is a prerequisite to the meaningful use of these assays in nonhuman primates.

In contrast to primates, other mammals do not lack 6‐pyruvoyl‐tetrahydropterin synthase and the metabolic pathway that favors the production of tetrahydrobiopterin rather than neopterin, resulting in lower levels of neopterin. However, neopterin can still be quantified in blood samples from various species, including dairy cattle, horses, sheep, llamas, dogs, cats, guinea pigs, mice, and rats (Aydın et al. 2025; Duch et al. 1984; Rokos et al. 1985; Stang and Koller 1998). Despite these studies, the potential of neopterin as a biomarker for cell‐mediated immune responses in these taxa remains largely unexplored.

## 4 Pathogens Associated With Increased Neopterin Levels

Neopterin production is closely associated with Th1‐type, cell‐mediated immune responses to intracellular pathogens and therefore represents a highly specific biomarker for these kinds of infections. While most intracellular pathogens are viruses, certain bacteria and protozoans, such as leprosy and malaria, also cause intracellular infections and therefore neopterin responses (see below). It is important to note that neopterin levels are usually not elevated in response to other pathogen types, particularly extracellular pathogens which typically elicit Th2‐type immune responses (but see below for certain exceptions). Therefore, low neopterin levels do not necessarily indicate that an individual is healthy or free from any infection. This limitation should be considered when interpreting neopterin levels.

### 4.1 Neopterin Responses to Viruses

All viruses stimulate a neopterin response (Wachter et al. 1989). Numerous viruses, including human immunodeficiency virus type 1 (HIV), hepatitis viruses (HBV and HCV), varicella‐zoster virus (chickenpox), measles, mumps, rubella, influenza, COVID‐19 (SARS‐CoV‐2), and Epstein‐Barr virus (EBV), have been shown to increase neopterin levels in human blood or urine during infection. Vaccination with live attenuated viruses, such as measles‐mumps vaccines, can also increase neopterin levels (Wachter et al. 1989). While all viruses increase neopterin levels, the magnitude of this response varies between pathogens. For example, average neopterin levels were higher during dengue fever than during measles, and lower during influenza infections (Chan et al. 2006). Therefore, neopterin is a highly sensitive biomarker of immune activation in response to viral infections. All tested viruses induced a measurable neopterin increase but the extent of this increase varies with specific viruses, possibly reflecting differences in the dynamics of immune system activation.

In nonhuman primates in wild and captive settings, infections are impossible to anticipate and challenging to monitor and notice. Therefore, controlled conditions, such as vaccinations or experimentally induced infections in biomedical research, provide an opportunity to monitor neopterin dynamics in response to known immune challenges. For example, several studies have documented increased urinary and blood neopterin levels following Simian immunodeficiency virus (SIV) and HIV infection in rhesus macaques (*Macaca mulatta*) (Fendrich et al. 1989; Higham et al. 2015; Stahl‐Hennig et al. 2002). Rabies booster vaccinations also led to increased neopterin levels in tufted capuchins (*Sabajus apella*) (Lucore et al. 2022). Additionally, the administration of human interferon beta to tufted capuchins resulted in increased neopterin levels in both urine and blood (Lahoz et al. 2009). This finding is consistent with in vitro studies in human macrophages and dendritic cells, in which interferon beta has been shown to stimulate neopterin synthesis—albeit to a lesser extent than IFN‐γ (Wirleitner et al. 2002).

To date, relatively few studies have investigated neopterin responses to naturally occurring diseases outbreaks in zoos or in wild populations. Neopterin levels increased in response to respiratory disease outbreaks in symptomatic zoo‐housed and wild bonobos (*Pan paniscus*), as well as in wild chimpanzees (*Pan troglodytes*) (Behringer et al. 2017; Kreyer et al. 2023; Thompson González et al. 2020; Wu et al. 2018). In a longitudinal study of wild immature chimpanzees, urinary neopterin levels were found to be unrelated to other disease markers (Löhrich et al. 2018). However, in a case study of a single immature chimpanzee, the onset of visible sickness behavior was followed by an increase in urinary neopterin levels (Löhrich et al. 2018).

### 4.2 Neopterin Responses to Protozoa

**Figure 2:**
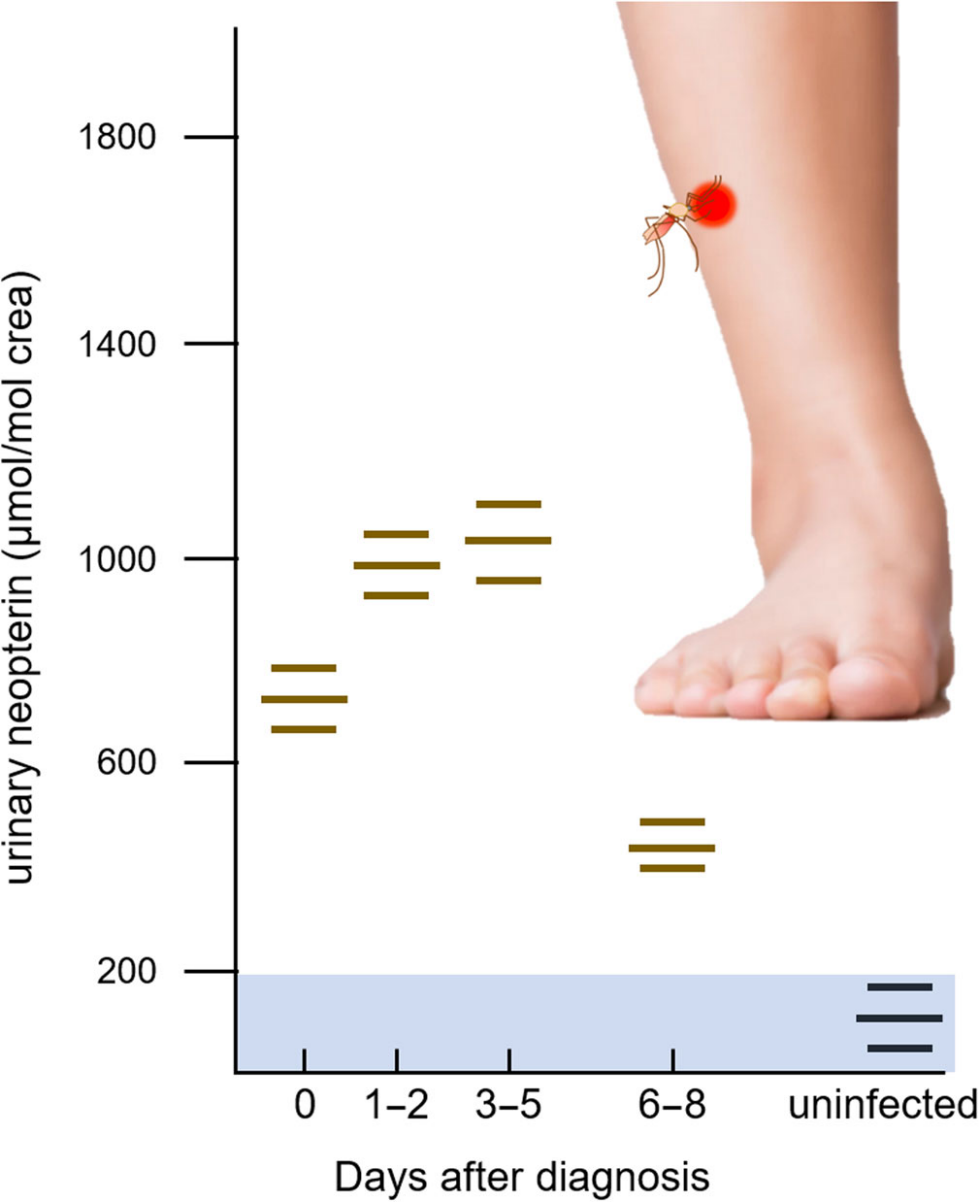
Schematic diagram of urinary neopterin levels from patients with acute falciparum malaria infection and uninfected control group. Horizontal bars represent mean ± SEM. The light blue area defines the normal range, calculated by the mean + 3 standard deviation of uninfected individuals. Modified after Brown et al. (1990).

Protozoa parasites that infect and multiply within host cells, such as *Plasmodium* spp. (the causative agent of malaria), stimulate Th1‐type immune responses similar to those elicited by viral infections (Figure 2). Consequently, these infections also lead to elevated neopterin levels in blood and urine (e.g., Berdowska and Zwirska‐Korczala 2001; Fuchs et al. 1988, 1989; Hamerlinck 1999; Rasmi et al. 2022; reviewed in Wachter et al. 1989, Table 3; Widner et al. 1999).

### 4.3 Neopterin Responses to Intracellular Bacteria

Like intracellular protozoa, bacterial pathogens that invade and replicate within host cells also lead to increased neopterin levels because they provoke the same Th1‐mediated immune responses. This includes bacteria responsible for diseases such as pneumonia, bacterial meningitis, brucellosis, and leprosy (Berdowska and Zwirska‐Korczala 2001; Eisenhut 2013). Overall, neopterin levels in response to bacterial infections are lower than those during viral infections, but certain intercellular bacteria, particularly *Mycobacterium leprae*, the causative agent of leprosy, can induce neopterin levels comparable to those found in viral infections (Denz et al. 1990; Eisenhut 2013).

### 4.4 Neopterin Responses to Extracellular Pathogen Infections

In contrast, host immune defenses against extracellular bacteria and many parasites are mainly mediated by humoral immune responses (details about these defense mechanisms are reviewed in Schechter et al. 2019). These include bacterial infections with *Bordetella pertussis*, *Streptococcus pneumoniae*, and *Neisseria gonorrhoeae*. As a result, these pathogens do not stimulate neopterin production in meaningful amounts (Wachter et al. 1989). Consistently, urinary neopterin levels remained unaffected by anthelmintic treatment in Barbary macaques (*Macaca sylvanus*) (N. Müller et al. 2017) and showed no association with larval tapeworm infection in wild geladas (*Theropithecus gelada*) (Schneider‐Crease et al. 2022).

Important exceptions to this pattern are infections with Gram‐negative bacteria. Lipopolysaccharide (LPS), a component of the cell envelope of Gram‐negative bacteria, induces inflammatory responses and stimulates neopterin release from macrophages, monocytes and dendritic cells either directly or by enhancing the effect of IFN‐γ (Schroecksnadel et al. 2010; Troppmair et al. 1988; Wirleitner et al. 2002). Accordingly, increased neopterin levels have been observed in septic patients compared to patients without sepsis, with higher neopterin levels correlating with increased severity and mortality rate (Strohmaier et al. 1987). Studies in nonhuman primates further supported this association. Baboons (*Papio* spp.) that were intravenously infused with live *Escherichia coli* to simulate sepsis showed a 20‐fold increase in blood neopterin levels within 24 h (Strohmaier et al. 1996). Similar patterns have been reported in wild chimpanzees, in which neopterin levels increased in response to severe physical injuries (Thompson González et al. 2020). Notably, these large increases in neopterin levels in relation to extracellular pathogen infections were reactions to severe and even systemic infections; smaller, locally restricted infections probably do not provoke neopterin production to such an extent.

These results highlight that although neopterin is a highly specific marker for Th1‐type immune responses to intracellular pathogens, it can also serve as an indicator of immune activation during severe extracellular bacterial infections. This is particularly relevant for systemic infections involving Gram‐negative bacteria. However, it is important to note that mild, localized extracellular infections likely do not induce neopterin production to a measurable extent.

In summary, neopterin is a robust and sensitive marker of cell‐mediated immune activation in response to intracellular infections under controlled and field conditions. Because neopterin also increases in response to infections by Gram‐negative bacteria, the interpretation of neopterin levels measured in wild primates should always consider the possibility of such infections. Furthermore, it is important to remember that stable or low neopterin levels do not necessarily imply an absence of immune challenges or infections, as many extracellular pathogens do not elicit an increase in neopterin. Rather, neopterin levels reflect certain aspects of immune responses to a specific group of pathogens, and are not an overall measure that reflects the complexity and diversity of immune responses to different pathogen types. Because of this specificity, neopterin can provide valuable insights into health and cell‐mediated immune dynamics in wild primate populations in relation to intracellular pathogens. For other pathogens, other biomarkers that reflect immune responses specific to these pathogens will be more appropriate.

### 4.5 Neopterin as a Marker for Inflammation and Chronic Diseases

A broad spectrum of noninfectious diseases and conditions involving inflammation are associated with changes in neopterin levels. Macrophages play a critical role in tumor surveillance and inhibition of malignant cell proliferation. Consequently, neopterin levels are a valuable prognostic marker in various cancers because they correlate positively with tumor stage and are associated with poor clinical outcomes (reviewed in Berdowska and Zwirska‐Korczala 2001).

Macrophages also play an important role in the pathophysiology of cardiovascular diseases. Increased neopterin levels have been reported in patients suffering from both acute and chronic coronary artery disease, reflecting the inflammatory component of these conditions (Berdowska and Zwirska‐Korczala 2001).

Neopterin is further elevated in several autoimmune disorders characterized by Th1‐mediated immune dysregulation. These include rheumatoid arthritis (Mangoni and Zinellu 2023), systemic lupus erythematosus (Bahrehmand et al. 2015), and autoimmune‐related thyroid diseases (Berdowska and Zwirska‐Korczala 2001).

Of particular relevance for primatologists is the application of neopterin as a biomarker for periodontitis, a chronic inflammatory disease of periodontal tissues that can ultimately result in tooth loss. Increased neopterin levels are observed in affected individuals, while successful nonsurgical treatment reduces neopterin levels (Heneberk et al. 2023). Given that dental health can be monitored remotely and potentially influences social and foraging behaviors in wild primates, neopterin could provide insights into the immunological cost of oral disease in natural populations.

Many of these chronic inflammatory processes probably contribute to age‐related increases in neopterin levels, which are discussed in more detail below.

## 5 Temporal Patterns of Neopterin Levels in Response to Acute Infection With Intracellular Pathogens: Implications for Sample Collection Timing

**Figure 3:**
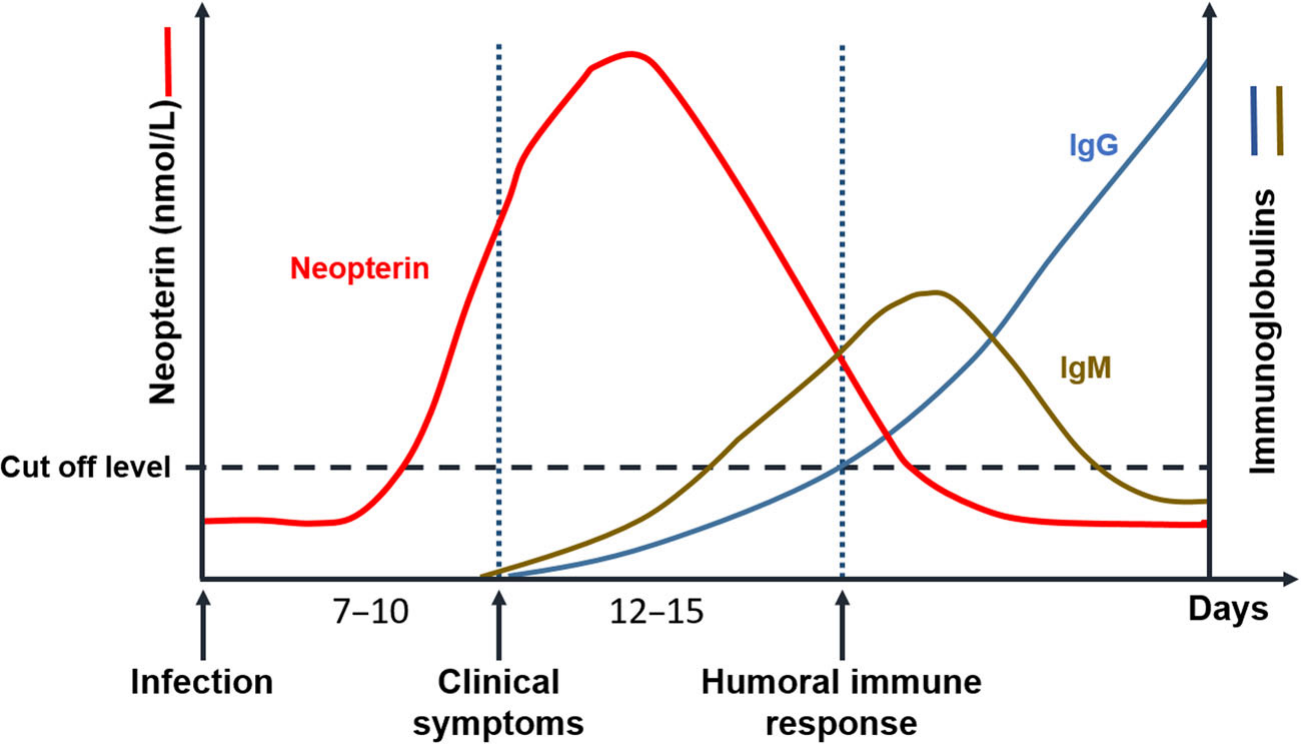
Schematic diagram showing the temporal course of neopterin levels in blood and detectability of specific antibodies during viral infections. Modified after Schennach et al. (1994).

The activation of cell‐mediated immune responses involving monocytes, macrophages, and dendritic cells in response to infections increases neopterin levels in blood and urine ~7–10 days after the onset of infection (Figure 3; Dittmer et al. 1995; Higham et al. 2015; Laliberté et al. 2023; Strohmaier et al. 1991). Following vaccinations, neopterin levels usually peak between Days 12 and 15 (Hamerlinck 1999; Wachter et al. 1989), and begin to decrease thereafter when antibody‐mediated responses become dominant (Figure 3; Eisenhut 2013; Hamerlinck 1999; Schennach et al. 1994). Therefore, the highest serum neopterin levels are observed just before infection‐specific antibodies are detectable (Fuchs et al. 1988). In dengue fever, neopterin level increase coincides with the onset of clinical symptoms (Chan et al. 2006), while in other viral infections, increased neopterin levels may precede the appearance of symptoms (Eisenhut 2013).

### 5.1 Considerations for Sample Collection Timing in the Field

It is crucial to be cognizant of these temporal patterns of neopterin production in relation to the time point of infection and the onset of symptoms for an appropriate timing and frequency of sample collection when neopterin is intended as a biomarker of acute infections or vaccination responses. Sampling too early, too late, or at too infrequent intervals may obscure meaningful patterns and hinder associations with behavioral or physiological variables.

In nonexperimental settings where the timing of infections cannot be anticipated, we recommend the following approach when acute neopterin responses are the primary research interest: samples should be routinely collected about 2–3 times/week. Retrospectively, samples that are associated with specific symptoms, behaviors, or other factors can then be selected for analyses, and compared to samples collected during asymptomatic times. An alternative approach could involve more frequent sampling of symptomatic individuals and reduced sampling during asymptomatic periods. We recommend collecting samples every second day to capture maximum levels. If the duration of increased neopterin levels as a measure of the overall immune response is of interest, it is also important to obtain 2–3 samples/week in the month after a potential infection. We are aware that such sampling regimes are resource‐intensive, particularly in field conditions, in which sample preservation and transport poses logistical challenges.

We are aware that these strategies depend on reliable detection of symptoms, which is often difficult in wild primates that are not routinely observed or cannot be monitored closely. In such cases, infections may go unnoticed and resulting samples could erroneously be categorized as asymptomatic despite increased neopterin levels. This introduces additional variance to the data and can complicate comparisons across individuals or time periods.

Interpreting neopterin data under natural, uncontrolled conditions remains challenging. Besides the timing of infection relative to sampling, the infection dose and severity may also influence neopterin level dynamics. Additionally, secondary bacterial infections occurring shortly after an initial viral infection may lead to a misattribution of symptoms and incorrect timing of sample collection (Grützmacher et al. 2018; Köndgen et al. 2017). Therefore, these complications highlight the importance of considering those limitations during the planning phase of study design.

## 6 Neopterin as a Predictor of Disease Severity and Outcome

Several studies have shown that neopterin levels are predictive of disease severity and outcome. Higher neopterin levels are indicative of more severe symptoms and prolonged disease duration in many viral infections. For example, in patients with dengue fever, neopterin levels were higher in patients experiencing a longer disease duration and more severe clinical symptoms, with peak levels corresponding to disease severity (Chan et al. 2006). During COVID‐19 infections, neopterin levels exceeding a certain threshold were predictive of fatal outcomes (death) with 64% specificity and 100% sensitivity (Chauvin et al. 2021). Similarly, during an outbreak of a viral respiratory disease in wild chimpanzees, urinary neopterin levels were higher in individuals that died compared to surviving individuals (Wu et al. 2018).

Neopterin also serves as a predictor for outcomes of severe bacterial infections. In patients with sepsis, higher neopterin levels were associated with poorer prognoses. For example, in cases of acute pancreatitis, individuals who died had higher neopterin levels than survivors, particularly on days 8 and 14 after disease onset. While survivors showed a gradual decrease in neopterin levels by day 14, they remained elevated in severe and fatal cases (Zhang et al. 2020).

Beyond infectious diseases, neopterin levels also predictive of outcomes of several chronic, noninfectious conditions. In patients with cardiovascular disease, higher neopterin levels were associated with greater disease severity and predicted adverse outcomes such as hospitalization or death (Lanser et al. 2019). Similarly, in older hip‐fracture patients, neopterin levels were a robust predictor of 1‐year mortality (Larsen et al. 2017).

Taken together, these findings highlight the potential of neopterin as a sensitive biomarker for assessing disease progression and predicting adverse outcomes of viral infections, sepsis, and chronic inflammatory diseases. However, it is important to recognize that neopterin reflects only specific aspects of the immune response, primarily cell‐mediated immunity, and does not capture the full complexity of the immune system and its responses.

## 7 Neopterin and the Microbiome

Changes in gut microbial communities affect health and disease by interacting with host genotype, diet, and other environmental factors that shape host physiology (Gomaa 2020; M. A. Martin and Sela 2013). The gut microbiota contributes to intestinal barrier defense against pathogens, regulates immune ontogenesis, and is involved in the process of immunosenescence (Amsterdam and Ostrov 2018; M. A. Martin and Sela 2013). Dysbiosis, a disruption of gut microbiota composition, has been associated with inflammatory states that are related to systemic gastrointestinal, metabolic, immunological, and neuropsychiatric disorders (Clemente et al. 2018). Therefore, several nonhuman primate studies have recently investigated gut microbiota in relation to host health, including work in rhesus macaques (Adriansjach et al. 2020), Assamese macaques (*M. assamensis*) (Anzà et al. 2023), geladas (Baniel et al. 2022), ring‐tailed lemurs (*Lemur catta*) (Bennett et al. 2016), bonobos (Hickmott et al. 2022), and black howler monkeys (*Allouatta pigra*) (Martínez‐Mota et al. 2022).

Only a few studies have investigated the relationship between neopterin levels and microbiome composition in nonhuman primates. In captive rhesus macaques, higher plasma neopterin and IFN‐γ levels were associated with increased abundance of Archaeal and Proteobacterial taxa, and decreased abundance of Firmicutes in older individuals compared to younger ones (Pallikkuth et al. 2021). In wild geladas, higher urinary neopterin levels were found in individuals with lower microbial alpha diversity, but there was no association with the relative abundance of specific microbial phyla, families, or genera (Schneider‐Crease et al. 2022). These relationships have been interpreted as evidence of age‐related inflammation linked to microbiome dysbiosis (Pallikkuth et al. 2021). However, they may also reflect independent, yet parallel age‐related physiological processes. Studies in younger children suggest that increased fecal neopterin may reflect normal intestinal macrophage activity during immune system development, rather than pathological inflammation driven by microbial composition (Pierce 2016).

These results reflect the mechanisms involved in the interaction between gut microbiome and neopterin production. Cells of the innate immune system, including macrophages, are located within the mucosal immune system of the gut and facilitate communication between the microbiome and host physiology (Thaiss et al. 2016). Overall, a balanced microbiome tends to reduce pro‐inflammatory processes, whereas dysregulation of the microbiome can enhance the activation of Th1 immune cells, potentially leading to chronic inflammation and autoimmune responses (Honda and Littman 2012). The microbial constitution of the microbiome contributes to the suppression of IFN‐γ production within the gut's innate immune system (Thaiss et al. 2016). Furthermore, intestinal macrophage activity can be influenced by LPS, an inducer of neopterin production (more details in Section 4.5). LPS is secreted by gut microbiota or can originate from bacterial gastrointestinal infections (Pierce et al. 2016). Therefore, associations between neopterin levels and gut microbiome diversity likely represent broader immunological processes that independently affect both neopterin production and gut microbiome composition. The current literature suggests that there are limited interactions between the gut microbiota and neopterin production. Other aspects of immunity might be better suited to investigate interactions between the microbiome and immune system.

## 8 Temporal‐ and Group‐Specific Patterns of Neopterin Levels

The immune system operates continuously to maintain homeostasis, and therefore macrophage activity and neopterin production are also present outside of acute or chronic disease states. In this section, we summarize several factors that have been found to contribute to temporal‐ and/or group‐specific patterns in neopterin levels.

### 8.1 Seasonal Changes in Neopterin Levels

Predictable, periodical variation in the prevalence of infectious diseases is a well‐known phenomenon in humans and animals, with notable effects on health, mortality, and consequently, population dynamics (Altizer et al. 2006; Nelson et al. 1995; ter Horst et al. 2021). These seasonal patterns can be caused by variation in the incidence of pathogens and parasites, by seasonal changes in immune functioning that affects host susceptibility, or are driven by other seasonally variable factors, such as behavior, that facilitate disease transmission (Altizer et al. 2006; L. B. Martin et al. 2008).

Seasonal changes in immune functioning may alter the ability to effectively respond to infections with specific pathogens. In regions where day length varies seasonally, melatonin is a major mediator of such immune variation (Hotchkiss and Nelson 2002; Walton et al. 2011). Melatonin synthesis increases in darkness and decreases during daylight. When nights are longer and days are shorter, the duration of melatonin production is prolonged, resulting in increased melatonin levels compared to periods with shorter nights and longer days. Under laboratory conditions, melatonin typically enhances immune functioning in vertebrates (Martin et al. 2008), with modulatory effects on T‐cell responses (Ren et al. 2017). In vitro experiments suggest that melatonin inhibits Th1 responses and reduces INF‐γ production, as indicated by lower serum levels of INF‐γ in melatonin‐treated mice (Ren et al. 2017). In line with these results, a study in rhesus macaques found that Th1‐type immune cells were less prone to synthesize INF‐γ in winter compared to summer (Mann et al. 2000). Consequently, neopterin levels in primates might also be expected to show seasonality, with lower levels during short‐day conditions.

We found only one study in humans that investigated seasonality in neopterin levels in relation to photoperiod. Contrary to expectations, blood neopterin levels were higher in winter than in the summer among adult Old Order Amish (Mohyuddin et al. 2017). Seasonal variations in immune functioning caused by changes in day length are probably diminished or masked by external conditions with stronger influences in individuals not living in tightly controlled laboratory settings. For example, increased time spent indoors during colder, shorter days may enhance the likelihood of pathogen transmission among humans, and therefore increase immune functioning during this time.

Most nonhuman primates live in regions with little seasonal variability in day length, and few studies have investigated seasonal variability in neopterin levels in these species. In bonobos, urinary neopterin levels showed annual periodicity, with the highest levels at the beginning of the short rainy season and lowest levels at the start of the long rainy season, but this pattern was not linked to changes in rainfall or temperature at the study site (Kreyer et al. 2023). In West African chimpanzees, urinary neopterin levels were highest during the long dry season when minimum ambient temperatures were lowest, and decreased at the end of the rainy season (Löhrich et al. 2018). Conversely, urinary neopterin levels in wild white‐faced capuchin (*Cebus imitator*) increased with temperature up to a thermal optimum of 30°C. Beyond this threshold, levels rapidly declined, suggesting a thermal performance curve for cell‐mediated immunity (Lucore et al. 2024). These findings indicate that seasonal variability in immune system functioning in primates is driven by other environmental factors than day length. For example, seasons with energetically demanding conditions may suppress immune responses, resulting in higher disease prevalence. Such conditions include environmental stressors such as low food availability, low ambient temperatures, and high predation pressure, as well as social stressors like increased competition during intense reproductive periods (Altizer et al. 2006; L. B. Martin et al. 2008; Nelson 2004; Nelson et al. 1995). In seasonally breeding mammals, pregnancy‐related changes in immune functioning (see below) might also cause seasonal patterns in pregnant individuals. To our knowledge, no studies have yet investigated these factors in relation to seasonally changing neopterin levels in primates.

Relationships between seasonal variation in rainfall, temperature, and neopterin levels likely reflect seasonal variation in the prevalence of certain pathogens. Directly transmitted viral infections, such as respiratory diseases or gastrointestinal viruses, follow seasonal patterns driven by interactions among virus/viral strain infectiousness, host contact patterns, pathogen survival duration, and host susceptibility (Fisman 2007). For example, respiratory syncytial virus and influenza are more prevalent during the rainy season (Shek and Lee 2003; Tamerius et al. 2013). Temperature and rainfall likely influence many of these factors associated with infection prevalence. Humans, for example, probably spend more time indoors when outside temperatures are very high or very low, increasing the likelihood of transmission.

Other infections require an intermediate host for transmission. These intermediate hosts are often arthropods like mosquitoes, ticks, flies, fleas, and lice. For such vector‐borne diseases, variation in vector and reservoir abundance, and vector biting rates, are additional factors in determining disease seasonality (Fisman 2007). For example, the presence of vectors that have aquatic larval stages, like mosquitoes transmitting malaria, depends on the availability of suitable water bodies, which can vary with rainfall seasonality (Altizer et al. 2006).

Together, these findings highlight the complexity of seasonal variation in immune functioning and suggest that multiple environmental and physiological factors shape neopterin dynamics in primates.

### 8.2 Diurnal Variation in Neopterin Levels

**Figure 4:**
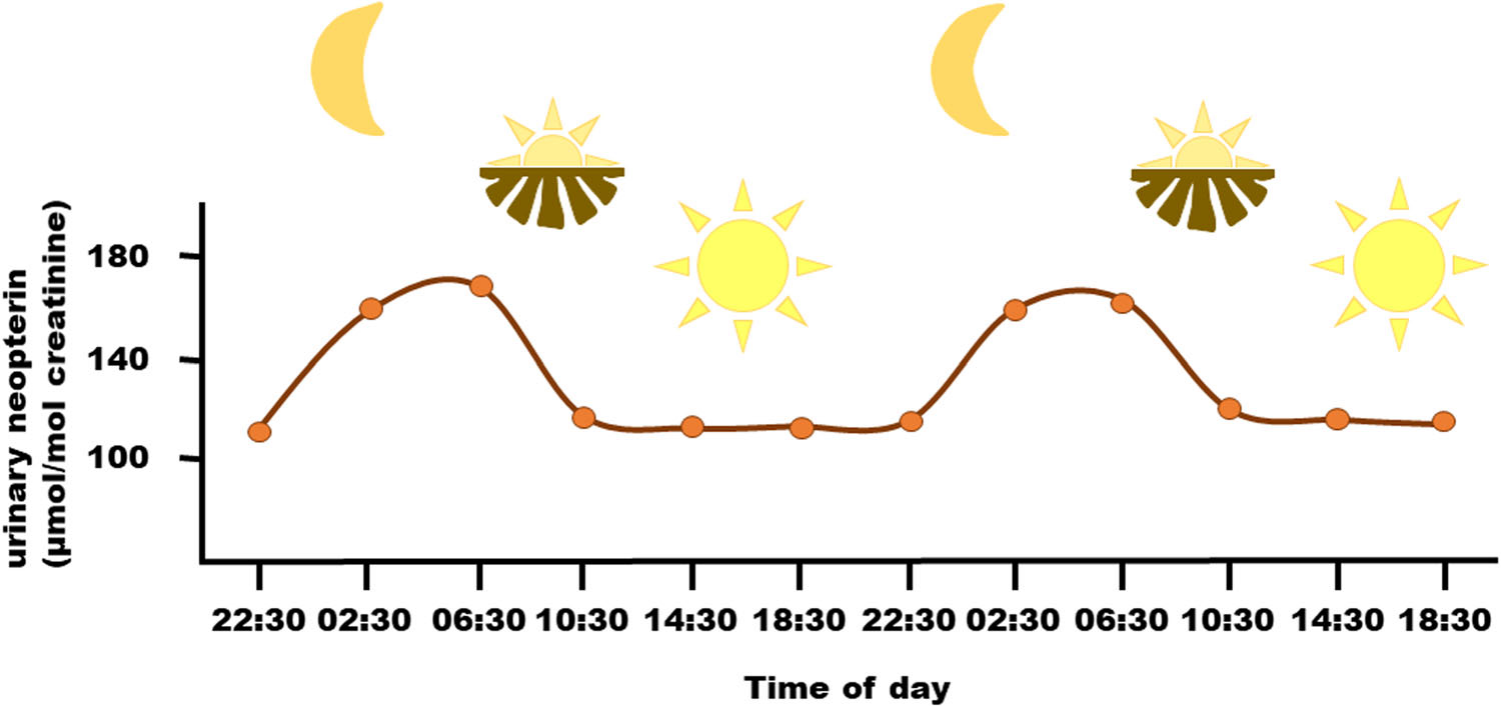
Schematic diagram of circadian rhythm in urinary neopterin concentrations over 3 weeks in five healthy men (adapted from Auzéby et al. 1988).

Many physiological markers follow circadian rhythms, which are predictable fluctuations that occur over ~24 h (Panda 2016). In humans, neopterin levels in blood and urine display a distinct circadian pattern (Figure 4): levels increase throughout the night, peak in the morning, and decline over the course of the day (Auzeby et al. 1988, 1989; Burton et al. 2016; Garcia‐Gonzalez et al. 2006). The light–dark cycle is probably the primary Zeitgeber (environmental cue) that regulates this pattern. Supporting this, urinary neopterin excretion patterns adjust in response to experimental shifts of the light–dark phase in humans (Schobersberger et al. 2007), and melatonin appears to play a regulatory role (Garcia‐Gonzalez et al. 2006). More information about melatonin's relationship with immune functioning can be found above in the section 8.1. Furthermore, dietary compounds, such as folate, and physical activity also contribute to daily neopterin fluctuation (Burton et al. 2016).

A similar circadian pattern of neopterin is probably present in nonhuman primates, though current findings are inconclusive. In line with human results, urinary neopterin levels decreased throughout the day in geladas (Schneider‐Crease et al. 2022), bonobos (Behringer et al. 2022, 2024), and chimpanzees (Negrey et al. 2021; Thompson González et al. 2020). However, some studies did not detect an association between neopterin levels and time of day, likely due to small sample sizes (Behringer et al. 2017), and potential cofounding factors such as young age (Behringer et al. 2021; Löhrich et al. 2018) or acute infections, which may obscure typical diurnal patterns (Behringer et al. 2017; Wu et al. 2018).

In clinical studies, neopterin's circadian variation has probably a minor impact on statistical outcomes, as the large effect sizes of neopterin production associated with diseases or infections surpass these comparatively small daily fluctuations (Burton et al. 2016). In other words, during illness, the immune‐related increase in neopterin levels is so pronounced that diurnal neopterin variation becomes negligible. In contrast, in studies where smaller effect sizes are expected, accounting for sampling time, either statistically or by standardizing sampling times, can be important to avoid the influence of this cofounding factor.

### 8.3 Age‐Related Differences in Neopterin Levels

In primates, immune functioning changes throughout life. Young individuals with naïve immune systems show a greater susceptibility to pathogens (Altizer et al. 2006; Dowling and Levy 2014), as do older individuals because of age‐related impairments in innate and adaptive immune responses, a process called immunosenescence (Castelo‐Branco and Soveral 2014; Channappanavar and Perlman 2020; L. Müller et al. 2019). These changes are also reflected in neopterin levels.

Numerous studies in humans and nonhuman primates found a relationship between neopterin levels and chronological age (from here on: age) (reviewed in Capuron et al. 2014; Schneider‐Crease et al. 2022). In humans, serum neopterin levels display a U‐shaped association with age, characterized by increased neopterin levels in both infants and older adults (Wachter et al. 1992; Werner et al. 1987). This U‐shaped pattern has also been observed in geladas (Schneider‐Crease et al. 2022), white‐faced capuchins (Lucore et al. 2022, 2024), and mandrills (*Mandrillus sphinx*) (Dibakou et al. 2020).

Studies excluding very young or very old individuals often report a linear relationship between neopterin levels and age, or no association at all. For example, studies focusing on younger individuals found decreasing neopterin levels with age, whereas research on older individuals report increasing levels with age in both humans (Catania et al. 1997; Diamondstone et al. 1994; Fahey et al. 2000; Frick et al. 2004; Ledochowski et al. 1999, 2001; Reibnegger et al. 1988; Spencer et al. 2010) and nonhuman primates (bonobo: Behringer et al. 2017, 2021; rhesus macaques: Cooper et al. 2022; rhesus macaques: Higham et al. 2015; tufted capuchin: Lucore et al. 2022; Barbary macaques: N. Müller et al. 2017). Nonhuman primate studies that lack representation of young and/or old individuals, or which include infected individuals, do not report age‐related differences in neopterin levels. This was found in several studies on chimpanzees (Behringer et al. 2019; Löhrich et al. 2018; Thompson González et al. 2020; Wu et al. 2018) and bonobos (Kreyer et al. 2023). These biases in the age or health status of sampled individuals likely obscure the age‐related differences observed in studies with broader age ranges and healthy individuals.

Overall, neopterin levels follow a consistent U‐shaped pattern with age across several primate species. This pattern appears to be a shared trait among humans and other primates and reflects underlying differences in immune system functioning that are characteristic of different life stages. This strong link between age and neopterin levels necessitates to account for age when analyzing data or when conceptualizing new studies. Comparative studies across populations and species focusing on immune system maturation and aging patterns will be useful to explore how environmental factors shape these processes (McDade 2003; McDade et al. 2016). It is important to note that most of these studies are cross‐sectional, comparing neopterin levels across individuals of different ages, rather than longitudinal, tracking changes within the same individuals over time. Longitudinal studies would be particularly valuable to investigate sex differences in immune aging and/or to assess how early‐life experiences influence later‐life immune functioning.

### 8.4 Sex‐Contextual Patterns in Neopterin Levels

Females and males often differ in the prevalence, severity, and pathogenesis of infectious, autoimmune, and age‐related diseases (Calabrò et al. 2023; Klein 2008; Nunn et al. 2009). Relevant factors that modulate immune functioning and that vary with sex/gender categories include genetic differences such as sex chromosomes, hormonal mediators such as estradiol, and environmental, social, and ontogenetic influences (e.g., Klein and Flanagan 2016; Mariencheck 2024; Nowak and Muehlenbein 2025; Oertelt‐Prigione 2012). As the studies referenced below usually rely on binary categorizations of sex—or, in case of human studies, sexgender—categories without necessarily specifying how individuals differ in immune‐relevant characteristics (DuBois et al. 2024; Springer et al. 2012), we use the labels as reported in the respective publication (e.g., male/female, men/women).

Cell‐mediated immune activity is typically more pronounced in females compared to males (Klein and Flanagan 2016; Nunn et al. 2009). However, numerous studies in humans and nonhuman primates consistently report no sex differences in neopterin levels. In humans, serum neopterin levels do not differ between men and women in most studies (e.g., Currie et al. 1994; Diamondstone et al. 1994; Werner et al. 1987). Likewise, no differences in urinary or fecal neopterin levels were found between males and females in several nonhuman primate studies, including Barbary macaques (N. Müller et al. 2017), rhesus macaques (Higham et al. 2015), geladas (Schneider‐Crease et al. 2022), chimpanzees (Behringer et al. 2019; Löhrich et al. 2018; Thompson González et al. 2020; Wu et al. 2018), bonobos (Behringer et al. 2017, 2021; Kreyer et al. 2023), and mandrills (Dibakou et al. 2019).

Exceptions to this pattern include findings of higher urinary neopterin levels in female rhesus macaques compared to males (Cooper et al. 2022, 2025) and higher neopterin levels in male mandrills and chimpanzees compared to females (Dibakou et al. 2020; Negrey et al. 2021). One human study also found a higher urinary neopterin‐to‐creatinine ratio in women than in men (Wachter et al. 1992). In primates, these differences have been attributed to factors such as the prevalence of chronic infections, life‐history stages, and sex‐specific physiology and behavior between the sex categories. For example, increasing testosterone levels during maturation may promote greater energetic investment in reproductive strategies and behaviors that indirectly affect immune functioning, potentially resulting in increased neopterin levels in males in some species or populations (Dibakou et al. 2020; Negrey et al. 2021). Testosterone also affects genes involved in infection resistance (Klein 2000; Zuk 2009), and elevated neopterin levels may reflect immune activation during periods of heightened competition, mate guarding, or aggression (Folstad and Karter 1992; Kurtz and Sauer 1999; Zuk 1990). Additionally, differences in the prevalence of chronic viral infections, such as higher rates of SIV in males, may contribute to increased neopterin levels in males (Dibakou et al. 2020).

Sampling bias related to life‐history stage could also influence observed sex differences. For example, studies sampling many females in late pregnancy may report higher average neopterin levels in females than in males (see below for more details). In cycling females, fluctuating estradiol and progesterone levels throughout the ovarian cycle can modulate immune functioning (Klein and Flanagan 2016), and biased sampling during specific cycle phases (e.g., luteal phase) could affect population‐level patterns. Behavioral changes related to the ovarian cycle may also play a role: increased social and sexual interactions or physical aggression during certain cycle phases can potentially elevate pathogen exposure and immune activity (Nunn et al. 2000). For example, wild female chimpanzees with maximum swellings, typically occurring around ovulation, have increased neopterin levels (Negrey et al. 2021).

Most studies do not find consistent differences in neopterin levels between sex categories in primates. It is possible that this is a result of the crude level of analysis these overly simplistic sex categories provide. Individuals grouped into binary categories of male and female can differ in a multitude of genetic, physiological, and behavioral characteristics that influence immune functioning (Nowak and Muehlenbein 2025). These characteristics do not necessarily group together within each binary sex category, and the variation of these characteristics within sex categories can be greater than between them (McLaughlin et al. 2023). Therefore, grouping individuals into binary categories might obscure differences that are associated with more specific sex‐related characteristics such as hormone levels. Additionally, context‐specific factors such as reproductive status, social behavior, infection risk, and sampling strategies may also contribute to variation within these categories and should be carefully considered when interpreting sex‐contextual patterns.

The relationship between neopterin and sex‐contextual characteristics warrants further research. Primates could be a good model system to test proximate and ultimate explanations for sex‐contextual differences in immunity. Operationalizing sex as a variable beyond overly simplistic binary sex categories, but contingent on the inherent complexity of sex as well as research context will be helpful to understand interactions with immune functioning in a more meaningful way (McLaughlin et al. 2023).

### 8.5 Immune Modulation and Neopterin Dynamics During Pregnancy

Mammalian pregnancy is an immunological challenge: the maternal immune system needs to protect both the mother and the developing fetus from potential infections while also remaining tolerant of foreign tissues within the uterine environment (Heerema‐McKenney 2018). Therefore, precise modulation of maternal immune activity is essential during embryonic implantation and throughout pregnancy to ensure a successful birth (Abu‐Raya et al. 2020; Morelli et al. 2015). Distinct pregnancy stages, which include implantation, fetal growth, and parturition, have different immunological requirements with complex maternal–fetal immune responses. While implantation and parturition are inflammatory processes, the environment for fetal growth is characterized by an anti‐inflammatory Th2‐type environment (Casazza et al. 2020; Mor et al. 2011). While balancing these complex immunological processes, the maternal–fetal immune system modulates cell‐mediated immune responses to avoid overstimulation of Th1 responses that are associated with unfavorable pregnancy outcomes, though these changes may also increase the susceptibility to certain diseases (Morelli et al. 2015).

Neopterin has been used as a tool to monitor maternal immune responses during pregnancy. In humans, neopterin levels are consistently increased in pregnant women compared to nonpregnant peers (Bichler et al. 1983; Boyunağa et al. 2005; Burns et al. 1999; Kronborg et al. 2007). Likewise, pregnant nonhuman primates of several species had higher neopterin levels than males or nonpregnant females (Behringer et al. 2024; Dibakou et al. 2020; Kreyer et al. 2023; Negrey et al. 2021; Thompson González et al. 2020). Moreover, neopterin levels fluctuate across pregnancy stages. The highest neopterin levels are found during the last trimester, with a sharp decline postpartum in both humans (Bichler et al. 1983; Boyunağa et al. 2005; Burns et al. 1999; Kronborg et al. 2007; Schennach et al. 2002; Schröcksnadel et al. 1996) and bonobos (Behringer et al. 2024, Figure 5).

**Figure 5:**
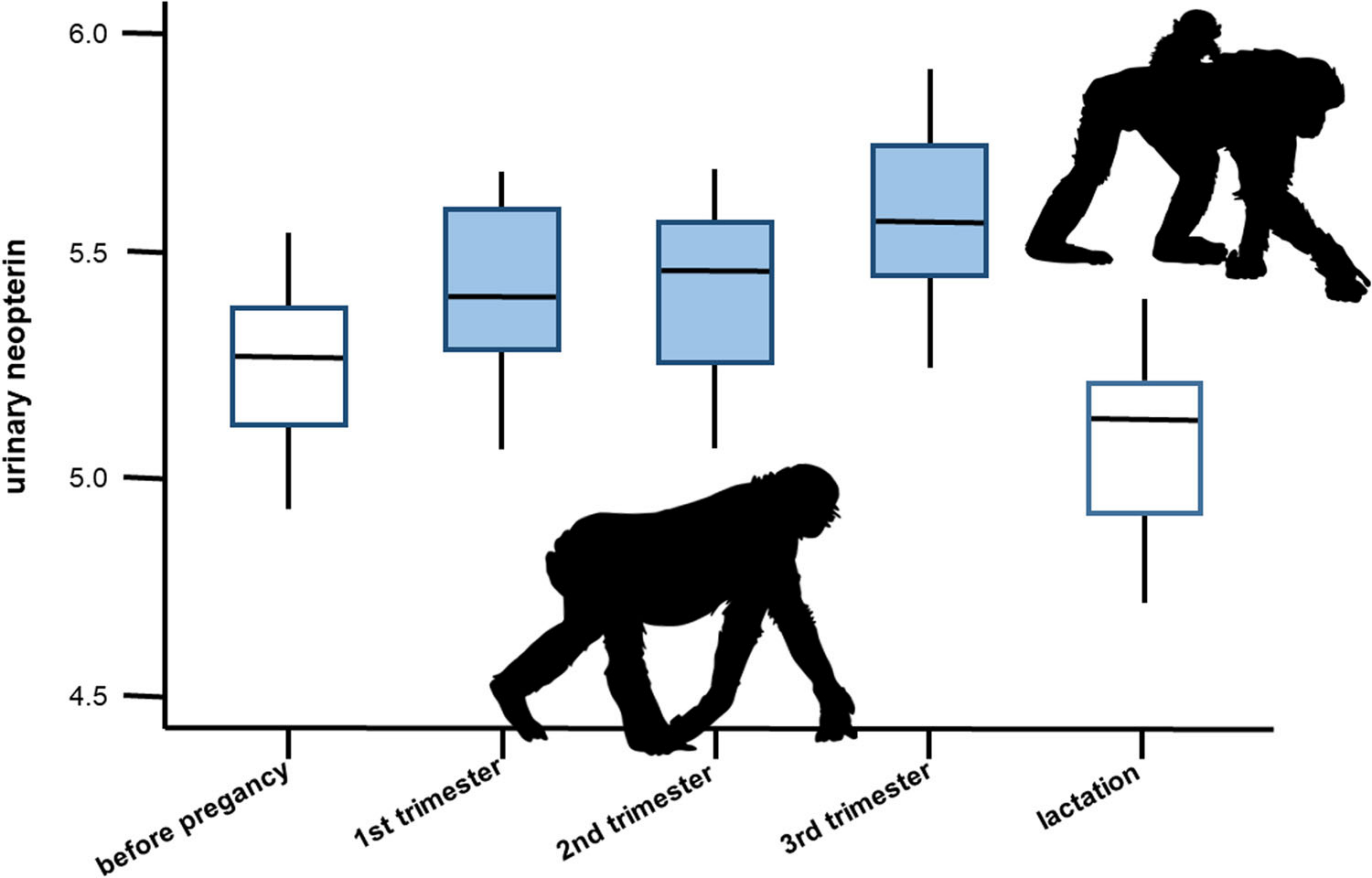
Schematic diagram of urinary neopterin levels of female bonobos before, during, and after pregnancy. White boxes indicate nonpregnant states, while blue boxes represent measurements from pregnant females (adapted from Behringer et al. 2024).

These robust patterns in neopterin production during pregnancy probably reflect maternal and fetal immune processes involving macrophage activation and inflammatory signaling that are not necessarily associated with the typical Th1‐type activation during infections. The placenta is rich in decidual and fetal macrophages (Aagaard‐Tillery et al. 2006), and IFN‐γ is crucial for successful pregnancies, but macrophage populations and sources of IFN‐γ change throughout different pregnancy phases (Casazza et al. 2020). For example, NK cells and macrophages make up the majority of decidual immune cells (the decidua is the modified mucosal lining of the uterus), while T cells are sparse (Orefice 2021). NK cells and macrophages are crucial during the implantation process and first trimester (Orefice 2021). These decidual NK cells secrete IFN‐γ, which promotes placental formation and maintenance of the decidua (Casazza et al. 2020), potentially explaining the increase in neopterin levels during the first trimester (Melichar et al. 2010).

As pregnancy progresses, NK cell numbers decrease, while placental macrophages of both maternal and fetal origin remain relatively stable, although their activation status changes from pro‐inflammatory to immunoregulatory (Mezouar and Mege 2020). Intriguingly, placental, fetal, and decidual macrophages themselves appear to be sources of IFN‐γ, particularly during the third trimester. This IFN‐γ production may contribute to fetal protection from bacterial infections before stimulating Th1‐dominated immune responses that are potentially detrimental for pregnancy (Mezouar and Mege 2020). It is conceivable that IFN‐γ produced by NK cells and macrophages during pregnancy is the reason for elevated neopterin levels in the absence of Th1 activation throughout pregnancy. Elevated neopterin levels during pregnancy may also serve to reduce increased oxidative stress, especially during later pregnancy stages (Fuchs et al. 1988; Gieseg et al. 2018; Toboła‐Wróbel et al. 2020).

Overall, neopterin levels throughout pregnancy probably reflect a number of different immune‐modulatory processes related to macrophages and IFN‐γ signaling that are unique to pregnancy and probably do not represent activation of Th1 pathways. Certain characteristics of these immunological processes during pregnancy are specific to humans, and it remains uncertain whether similar mechanisms are present across nonhuman primates (Casazza et al. 2020; Mezouar and Mege 2020). If neopterin reflects components of these pregnancy‐related immune processes, it may serve as a valuable tool for comparative investigations across primate taxa.

## 9 Practical Guidelines: How to Measure Neopterin

Neopterin can be measured with accessible methods in a range of sample matrices. This flexibility highlights its suitability as an immune marker in primate studies, where blood sampling is often restricted by practical reasons and ethical considerations. Each method and sample matrix offers distinct advantages and disadvantages, and the optimal choice depends on research questions, study species, feasibility of sample collection, and availability of laboratory infrastructure. In this section, we summarize these aspects and provide practical guidelines for the collection, storage, and quantification of neopterin.

### 9.1 Suitability of Different Sample Matrices for Neopterin Analysis

Neopterin can be quantified in blood, urine, saliva, and fecal samples. The selection of a suitable sample matrix for a given research project depends on: (1) the biological relevance of neopterin levels in this matrix, (2) the analytical characteristics of the matrix, (3) the required sampling frequency, (4) traits of the study population, and (5) storage and logistical requirements. Below, we summarize the important aspects of each sample type to support informed decision‐making in future research projects.

#### 9.1.1 Advantages and Limitations of Neopterin Quantification in Blood Samples

Neopterin can be reliably quantified in serum and plasma and is routinely measured in human clinical settings, where standardized collection and processing of samples ensure consistent results. Using blood samples allows for the simultaneous assessment of other immune biomarkers (e.g., cytokines, C‐reactive protein, or specific immunoglobulins) that may not be quantifiable in other matrices (Higham et al. 2015). Blood also facilitates the measurement of hormones and oxidative stress markers. Commercially available ELISA kits enable direct quantification of neopterin in blood without prior extraction steps, making the method time‐ and cost‐efficient, and well‐suited for larger sample sets.

However, blood sampling has several limitations. Neopterin levels in blood reflect the neopterin production at the exact time of sampling and are therefore sensitive to diurnal fluctuations (see above). In these cases, the time of day is an important variable to consider when comparing measurements within and/or between individuals.

In nonhuman primates, collecting blood samples is often stressful, as it typically requires restraint and sedation. These procedures limit sampling frequency because they introduce health and safety risks, and other welfare concerns for the animals. Certain individual characteristics can prohibit blood sampling because of the stress and potential risks involved, for example, those of young or advanced age, pregnant individuals, or those caring for dependent offspring. The need for veterinarians and other trained personnel to perform the necessary procedures are further logistical challenges. When working with wild primates, these factors make blood sampling unfeasible in most circumstances.

#### 9.1.2 Advantages and Limitations of Neopterin Quantification in Urine Samples

Neopterin can be reliably quantified in urine, and levels measured in urine correlate with those in blood. A constant ratio between neopterin and 7,8‐dihydroneopterin in both serum and urine further supports the reliability and suitability of urine samples (Fuchs et al. 1988; Higham et al. 2015; Wachter et al. 1992; Westermann et al. 2000). This consistency indicates that urinary neopterin provides comparable information on cell‐mediated immune activation as blood‐derived measures. Urinary neopterin can be measured with commercially available ELISA without prior extraction procedures.

A major advantage of urine sampling is that it can be performed without capture, handling, and/or sedation. This allows frequent, longitudinal sampling in both captive and field settings. Depending on the species and the environment, urine collection often provides larger sample volumes than serum collection, facilitating analyses of neopterin and other biomarkers in the same sample. This might also be relevant when working with small‐bodied species such as common marmosets (*Callithrix jacchus*) or mouse lemurs (*Microcebus*), where the volume of blood that can be sampled is limited.

Unlike blood samples, urine concentrations are influenced by an individual's hydration status. Animals maintain a controlled internal milieu regarding water and electrolytes by osmoregulation. The amount of water excreted by the kidneys depends on factors such as variation in fluid and salt intake, aldosterone secretion, and evaporative thermoregulation by panting and sweating (Anestis et al. 2009; Costa et al. 2013; Wachter et al. 1992). To account for these fluctuations, biomarker concentrations in urine are normalized using measures of urine concentration. For spot urine samples, creatinine and SG are the most commonly applied correction factors to standardize urinary neopterin concentrations (e.g., Alessio et al. 1985; Cone et al. 2009; Miller et al. 2004).

Creatinine, a byproduct of muscle and protein catabolism, is excreted in urine at a consistent rate in healthy individuals (Jackson 1966) and is widely used to standardize urinary neopterin levels across individuals and time. Creatinine is stable during long‐term storage under frozen conditions, but is susceptible to degradation when stored at room temperature (RT) and exposed to repeated freeze–thaw cycles (Daniels 2013; Garde et al. 2003; Heistermann and Higham 2015; Higham et al. 2020; Remer et al. 2014; Saude and Sykes 2007; Spierto et al. 1997). This method is regularly applied in human neopterin studies. Therefore, if direct comparison with such data are intended, urinary neopterin levels should be corrected for urinary creatinine concentration (Alessio et al. 1985; Miller et al. 2004).

But creatinine production is dependent on individual differences in morphology and lifestyle. Differences or changes in muscle mass can bias creatinine concentrations, and thus, neopterin levels corrected for creatinine. This is important to consider for longitudinal studies monitoring individuals from infancy to adulthood, or for comparison between groups that differ systematically in body size and muscle mass, such as sexually dimorphic species or flanged and unflanged orangutan males (Alessio et al. 1985; Carrieri et al. 2000; Donadio 2017; van Niekerk et al. 1963). Additionally, factors such as diet, diurnal rhythms, population differences, renal functioning, and health status can influence creatinine levels in more or less predictable ways (Barr et al. 2005; reviewed in Brosnan and Brosnan 2016; Crim et al. 1975; Curtis and Fogel 1970; Miller et al. 2004; Oterdoom et al. 2009; Sallsten and Barregard 2021; Wettersten et al. 2021). In studies of wild primates, these uncontrollable factors may introduce unexplained variation in the data that might mask biological meaningful patterns.

An alternative to creatinine is the use of SG (also called relative density), a well‐established correction method in both human and nonhuman primate studies (Anestis et al. 2009; Athanasiadou et al. 2018; Emery Thompson et al. 2012; Imran et al. 2010; Krief et al. 2005). SG is a dimensionless ratio of urine density relative to water density measured by refractometry. It is a cost‐effective and time‐efficient method that can be implemented in field settings and by personnel with limited laboratory training (Pradella et al. 1988). Neopterin concentrations are corrected using SG values by using a formula based on the Levine–Fahy equation, which incorporates the population mean SG value (Cone et al. 2009; Levine and Fahy 1945; Miller et al. 2004). Urine samples with SG values below 1.003 should be excluded, as low‐density samples can artificially inflate analyte concentrations (Cone et al. 2009; Emery Thompson et al. 2012; Goldberger et al. 1995). Notably, SG measurements often remain above this exclusion threshold even when creatinine concentrations are low and cannot be used (< 0.05).

Ideally, urinary SG should be measured at 20°C; although this is not always feasible in field conditions without climate control (Wardenaar et al. 2021). Like creatinine, SG remains stable in samples under long‐term frozen storage and for up to 48 h at RT (Neumann et al. 2020). In contrast to creatinine, SG is also stable through repeated freeze–thaw cycles (Anestis et al. 2009). Although SG and creatinine values are highly correlated (Carrieri et al. 2000; Newman et al. 2000; White et al. 2010), SG corrected analyte levels are less sensitive to confounding influences such as muscle mass, hydration status, and dietary variation (Carrieri et al. 2000; Suwazono et al. 2005; White et al. 2010).

In summary, SG is an efficient and reliable alternative—or complementary—method to creatinine correction, depending on the analyte, study design, and demographics of the study population. SG correction is particularly advantageous in comparative studies involving individuals with varying physiological or lifestyle profiles, as it reduces the influences of confounding factors and improves the comparability of analyte levels across groups.

#### 9.1.3 Neopterin Quantification in Saliva and Fecal Samples

Although saliva and feces are technically suitable for neopterin measurement, their application in primate studies remains limited to specific contexts. Saliva and fecal neopterin levels are not correlated with neopterin levels measured in blood (Higham et al. 2015; Ikemoto et al. 2021), and probably represent more local immunological processes of the oral cavity and the gut than systemic ones.

Neopterin can be detected in human saliva (Ikemoto et al. 2021; Katoh et al. 1989), and is used as a diagnostic tool in the field of periodontics (Mahendra et al. 2014; Ozmeriç et al. 2002; Vrecko et al. 1997). While such applications are well‐established in humans, the biological interpretation of salivary neopterin is less developed for nonhuman primates.

Fecal neopterin has shown diagnostic potential in certain clinical contexts, for example, during acute gastritis, active or inactive Crohn's disease, or active ulcerative colitis (Husain et al. 2013; Ledjeff et al. 2001), but not for *Giardia* infection in humans (Campbell et al. 2004). One study in younger children found that fecal neopterin levels possibly indicate intestinal macrophage activity in relation to microbiome composition or immune system maturation activity rather than inflammatory processes (Pierce 2016; Thaiss et al. 2016). Neopterin has been successfully quantified in fecal samples of nonhuman primates (Dibakou et al. 2019; Higham et al. 2015), but fecal neopterin levels are independent of serum neopterin levels (Higham et al. 2015). Fecal neopterin levels were not associated with SIV infection (Higham et al. 2015) or intestinal protozoan and nematode parasitism (Dibakou et al. 2019).

To summarize, the usefulness of salivary and fecal neopterin measurements is probably limited to very specific questions as they appear to primarily reflect localized mucosal immune responses. This limits their application for assessing systemic immune activation in nonhuman primate studies and for broader ecological or physiological research questions.

### 9.2 Sample Storage and Handling

Best practices in sample storage and handling are essential to ensure accurate measurement of neopterin and to minimize biases arising from inconsistent sample management. Many nonhuman primate studies are conducted in settings where controlled conditions for sample storage and handling are often challenging. Given that many primate studies take place in remote field locations, we recommend Heistermann and Higham (2015) for a comprehensive overview for processing and storing neopterin samples under field conditions. In this section, we highlight key issues affecting neopterin stability that are important to consider in all conditions.

#### 9.2.1 Thawing and Freezing Cycles

Frequent freeze–thaw cycles have been shown to affect neopterin levels in biological samples. In humans, serum neopterin levels are particularly sensitive to repeated freeze–thaw cycles (Laich et al. 2002). Similarly, studies on bonobos and rhesus macaques have demonstrated increased urinary neopterin levels with additional freeze–thaw cycles, suggesting that such handling may introduce measurement artifacts (Behringer et al. 2017; Heistermann and Higham 2015). To avoid this effect, it is recommended to minimize or—ideally avoid—repeated freeze–thaw cycles wherever possible (Behringer et al. 2017).

When sample volumes permit, dividing samples into aliquots prior to freezing is recommended, as this allows independent analyses without the need to repeatedly thaw the entire sample. This approach is particularly useful when several biomarkers will be quantified in the same sample. If aliquoting is not an option, the number of freeze–thaw cycles each sample undergoes should be recorded so that these effects can be statistically controlled for during data analysis.

#### 9.2.2 Neopterin Stability During Storage

The stability of neopterin is influenced by storage conditions and sample matrix. Therefore, it is important to evaluate its stability across different biological matrices, storage conditions, and time frames, especially during study planning and development of optimal sampling strategies.

Neopterin levels are stable during long‐term storage when samples are kept frozen. In humans, both serum and urinary neopterin levels remain stable for at least 6 months when stored at −20°C (Laich et al. 2002). Similarly, neopterin levels remain stable up to several years in frozen urine and fecal samples from nonhuman primates (Behringer et al. 2019; Dibakou et al. 2019, 2020; Heistermann and Higham 2015; Thompson González et al. 2020).

Short‐term storage at RT does not seem to affect neopterin stability. In human serum and urine, neopterin levels are stable for up to 2 days at RT and for up to 2 weeks at 4°C (Laich et al. 2002; Wachter et al. 1989, 1992). In rhesus macaques, urinary neopterin remained stable for up to 4 months at RT in preserved and unpreserved urine samples. However, creatinine degradation over time in unpreserved samples can lead to artificially elevated neopterin‐to‐creatinine ratios and greater disruption of sample rank order with prolonged storage (Heistermann and Higham 2015). When urine samples have to be stored at RT, using SG rather than creatinine to correct for urine concentration may be an option. If this approach is taken, it is essential to verify that SG remains stable at RT. Alternatively, SG can be measured immediately upon sample collection using handheld refractometers, which can also be used in field settings.

Storing urine samples on filter paper at RT leads to increased neopterin concentrations, with increasing levels with longer storage durations (Heistermann and Higham 2015; Schneider‐Crease et al. 2022). Despite this, dried urine samples generally maintain a consistent rank order relative to controls, indicating that they still reflect biologically meaningful patterns despite absolute concentration changes (Schneider‐Crease et al. 2022).

Overall, neopterin demonstrates considerable stability in urine and serum across different storage temperatures. However, changes in creatinine concentration during storage at RT can introduce variability in neopterin values when creatinine is used to correct for urine concentration. While standard laboratory protocols ensure sample stability in most matrices, alternative storage methods, such as filter paper‐based techniques, require validation to maintain data integrity. It is also important to note that most degradation experiments are usually conducted under controlled laboratory conditions, whereas field storage environments may experience fluctuations in temperature and humidity, potentially accelerating degradation. Additionally, urine samples from wild‐living primates may contain dietary compounds absent from samples of captive individuals, which could potentially affect neopterin stability under suboptimal storage conditions. Freezing samples until analysis remains the recommended best practice for all sample types. When working with wild populations, it is strongly advised to test neopterin stability using pilot samples collected from the same population under similar storage conditions that are anticipated in the field and during transport.

#### 9.2.3 Neopterin Susceptibility to Light

Neopterin is susceptible to photodecomposition by long‐wave ultraviolet light (UVA) (Laich et al. 2002; M. Müller et al. 1991; Wachter et al. 1992). Exposure to daylight at RT (~25°C) in closed containers resulted in rapid degradation of neopterin, with almost complete conversion to pterin‐6‐carboxylic acid within hours due to photooxidation (Fuchs et al. 1982). To ensure accurate and reliable measurements, minimizing sample exposure to UVA light during all stages of sample handling is essential.

Several practical strategies can effectively reduce light‐induced degradation. For example, wrapping sample tubes in aluminum foil provides a physical barrier against light exposure (Wachter et al. 1992). Many manufacturers offer black or amber colored microcentrifuge tubes that protect sensitive samples from light degradation. In field settings, storing them in light‐proof containers such as thermos flasks until they can be safely stored back at camp should be standard practice. In laboratory environments, neopterin remains stable under artificial light conditions (Behringer et al. 2017; Laich et al. 2002) but analyses should be performed away from direct natural light sources, such as windows. Integrating these preventative measures into standard operating procedures and protocols helps to preserve neopterin stability.

## 10 Neopterin Quantification Methods

Neopterin can be readily quantified using ELISA in serum, plasma, urine, feces, and other biological matrices without requiring extraction procedures prior to analysis (Gieseg et al. 2018; M. Müller et al. 1991). Commercially available kits facilitate high‐throughput analysis, are user‐friendly, and are suitable for people with limited laboratory experience. Furthermore, the equipment necessary for ELISA is more widely accessible than that required for high‐performance liquid chromatography (HPLC; see below). Therefore, ELISA is the most widely used method for neopterin quantification (Agilli et al. 2012; Gieseg et al. 2018; Hamerlinck 1999; Werner et al. 1987; Westermann et al. 2000).

Radioimmunoassays (RIA) have also been used to quantify neopterin. This technique is based on similar immunological principles as ELISA but utilizes radioactive tracers. Due to increasing costs of tracers, the need for dedicated isotope laboratory infrastructure and safety protocols, and the widespread availability of ELISA kits, RIA is now rarely used for neopterin analysis. Furthermore, RIA has been shown to produce limited accuracy when analyzing urinary neopterin (Werner et al. 1987).

HPLC offers an alternative to ELISA/RIA technologies (Gieseg et al. 2018). Unlike ELISA or RIA, HPLC is highly compound‐specific and allows for the separation and quantification of structurally similar pterin derivatives that ELISA kits cannot discriminate (Agilli et al. 2012; Fuchs et al. 1982; Krcmova et al. 2011; Lindsay et al. 2014; Wachter et al. 1992). For studies aiming to quantify pterin derivatives for which no ELISA is available, such as 7,8‐dihydroneopterin and 5,6,7,8–tetrahydropterin, HPLC offers a valuable alternative (Gieseg et al. 2018).

However, HPLC methods are usually less sensitive than ELISA, which is problematic for matrices with low neopterin levels, such as plasma, in which levels are ~200‐fold lower than in urine (Hamerlinck 1999). Another disadvantage is the often labor‐intensive and time‐consuming sample preparation required to remove proteins and other interfering substances before analysis (M. Müller et al. 1991; Murtagh et al. 2013). Nevertheless, because HPLC columns have become relatively affordable compared to sample preparation costs, and given the low protein content and high neopterin levels in urine, diluted urine can often be injected directly for quantification (Lindsay et al. 2014). When HPLC instrumentation and expertise are available, it is a viable and precise alternative to ELISA for the quantification of neopterin and other derivatives.

Regardless of the quantification method used, researchers have to validate biomarker quantification procedures for each new species and sample type. This assures that measurements and results are comparable across studies, and that results are biologically relevant. The same principles apply for the validation of neopterin measurements as they do for other biomarkers. Rigorous method validation includes tests for specificity, sensitivity, and reproducibility of measurements (Behringer et al. 2017; Goymann 2005; Palme 2005; Wielebnowski and Watters 2007). Ideally, these tests are performed before starting a study, so potential problems can be addressed beforehand.

## 11 Summary

Neopterin is a valuable biomarker reflecting Th1‐mediated immune system activation, with broad applicability in primatology. Given its specificity regarding immune system functioning and pathogen type, neopterin is a promising biomarker for understanding trade‐offs in primate cell‐mediated immune functioning during intracellular infections but the interpretation of results requires careful consideration of the complexity of immune system responses to different kinds of pathogens. Neopterin is well‐suited for ecoimmunological research in both captive and wild primate populations. It is stable and can be readily quantified in different sample matrices, of which urine has the greatest potential for most applications in primatology. Our recommendations for best practices underscore the importance of accurate measurement methods, appropriate sample storage, and the need for methodological validation and standardization across studies. Furthermore, neopterin levels vary with several environmental and life‐history factors at the individual and population level, such as age, reproductive state, sex‐related variation, temperature, food availability, and pathogen pressure. Future research should focus on expanding longitudinal studies to explore these complex interactions of neopterin with age, sex, reproductive state, and environmental variables to further our understanding of primate immunity and its evolutionary impact.

## 12 Integrating Neopterin Into Evolutionary, Ecological, and Welfare Research

Practical methods to monitor specific aspects of immune functioning in captive and wild primates are needed, because the testing of many hypotheses in evolutionary ecology and ecoimmunology requires the quantification of immune system activation. Neopterin is a well‐established biomarker of cell‐mediated immune responses that can be measured in urine, and its application in different nonhuman primate species has already produced valuable insights.

We anticipate that neopterin will be increasingly incorporated into studies across different primate species and ecological contexts. It is well‐suited for testing hypotheses such as the immunocompetence handicap hypothesis and enhanced female immunocompetence in wild primates (Mariencheck 2024; Muehlenbein and Bribiescas 2005; Nowak and Muehlenbein 2025), as long as it reflects Th1‐mediated immune system activation. Furthermore, neopterin could be a valuable tool for disease surveillance and early detection of viral outbreaks, such as largely asymptomatic yellow fever infections in African primates, supporting OneHealth initiatives (Nederlof et al. 2025).

Another promising topic for research involves the relationship between neopterin levels and sickness behavior, a coordinated set of behavioral changes during infection (French et al. 2023). Inflammatory processes and cell‐mediated immune activation are associated with increased neopterin levels, and both mechanisms are suspected to be involved in the expression of sickness behavior in humans (Maes et al. 2012). Therefore, neopterin might be a tool to explore sickness behavior in both humans and nonhuman primates from evolutionary and anthropological perspectives (Shattuck and Muehlenbein 2015, 2016).

Among the behavioral responses to infection, sleep is a key component due to its bidirectional relationship with immune functioning. Sleep is affected during infections, but it is also central to immune competence and disease susceptibility, thereby impacting overall health and fitness. Mounting an effective immune response to infections or vaccination is dependent on sleep quality and sleep disturbance, and deprivation impairs Th1‐type responses and increases pro‐inflammatory cytokines such as IFN‐γ (Besedovsky et al. 2019; Garbarino et al. 2021). For primates, maintaining adequate sleep or meeting increased sleep demands in response to infections might be challenging when faced with simultaneous ecological and social pressures (Loftus et al. 2022). Thus, neopterin could be used to evaluate these trade‐offs in evolutionarily relevant contexts. Our review provides the theoretical and practical guidance for integrating neopterin into these research areas. Collecting data on variation in immune functioning across species with unique social and ecological contexts helps to answer questions and test hypotheses in evolutionary and applied ecoimmunology. A recent study of wild juvenile blue monkeys exemplifies how neopterin can reveal complex energetic trade‐offs in the wild. Cell‐mediated immune responsiveness, indicated by increased neopterin levels, was not impairing long‐term growth, but was contingent on individual body condition and energy balance, rather than external social or environmental variables (Thompson González et al. 2025).

In the context of disease resistance and tolerance, ecoimmunology offers a framework for understanding patterns of resource allocation and potential trade‐offs with other life‐history traits (Rauw 2012). While resistance to pathogens eliminates or prevents infection, tolerance modulates the severity of illness without necessarily eliminating the pathogen. For example, a comparative genomic study found differences in innate immune specificity and sensitivity between apes and haplorrhines that might be related to species‐specific life‐history strategies (Hawash et al. 2021). Reaction norm approaches, which are increasingly applied to investigate hormonal phenotypes, are a promising method for characterizing individual immune responses along gradients of internal or external conditions (Malkoc et al. 2022; Sonnweber et al. 2023). Given that the reaction norm approach requires repeated sampling of individuals, urinary neopterin is especially suitable for such research, allowing detailed examination of the costs, limitations, and adaptive value of diverse immune strategies.

Such research has not only evolutionary relevance but also conservation implications. The role these differences in immune strategies play in an individual's ability to adapt to changing environmental conditions is important for improving conservation efforts. Identifying drivers of immune phenotypes, and their links to disease susceptibility, fitness, and mortality, can help to predict outcomes and success of mitigation measures in applied conservation efforts (Ohmer et al. 2021). In this context, reaction norm approaches will be critical in linking individual immune strategies to patterns at the population or even species level, to improve conservation efforts in a world defined by anthropogenic and climate change (Taff et al. 2024). This knowledge will also be critical for integrative conservation approaches like OneHealth (Cunningham et al. 2017; Gillespie et al. 2008).

Finally, the complex link between immune functioning and animal welfare also remains understudied, despite known bidirectional effects between immunity and affective states (Düpjan and Dawkins 2022). Depressive symptoms such as anxiety and anhedonia are mediated by inflammatory immune processes involving cytokines like IFN‐γ (Maes et al. 2012), which accelerates tryptophan degradation and reduces serotonin synthesis. Neopterin levels correlate with tryptophan degradation, suggesting these processes might underly the development of depressive disorders (Leonard and Maes 2012; Widner et al. 2002). Therefore, neopterin might be an interesting candidate as a physiological marker to assess aspects of affective states in captive and wild primates that are associated with health and oxidative stress (Beaulieu 2024a, 2024b).

It offers cross‐species comparability, well‐established and accessible methodologies, and cost‐effective analyses suitable for larger sample sizes. Further neopterin measurement in urine is a sampling method that does not require catching and handling, or greater disturbance of habituated individuals. These advantages make neopterin research suitable for longitudinal, multifactorial, and replicated approaches in animal welfare contexts (Beaulieu 2024a). However, before neopterin could be used as a welfare indicator, it must be thoroughly validated as a proxy of subjective animal welfare. Causal indicators that affect welfare, such as disease, can be used to validate effect indicators of welfare states such as neopterin (Browning 2023). In captivity, the knowledge about how neopterin relates to affective states could then be used to improve welfare in captivity, to monitor the health and aging processes of individuals and help guide informed management decisions. In the wild, neopterin might help us to better understand affective states in natural conditions.

We hope that this review encourages researchers to incorporate neopterin into their research fields to advance our knowledge of cell‐mediated immune responses in nonhuman primates in both captivity and natural settings. We are also convinced that anthropologists and human biologists can benefit from using this biomarker to study immune functioning in diverse groups of people and environments. Integrating evolutionary, ecological, and social perspectives into immunological research will greatly advance our understanding of these complex physiological processes.

## Author Contributions

**Verena Behringer:** conceptualization (equal), visualization (lead), writing – original draft (lead), writing – review and editing (equal). **Caroline Deimel:** conceptualization (equal), writing – original draft (supporting), writing – review and editing (equal).

## Ethics Statement

All procedures were entirely noninvasive and complied with the ASAB/ABS Guidelines for the Use of Animals in Research (https://www.asab.org/ethics). The research adhered to the American Society of Primatologists (ASP) Principles for the Ethical Treatment of Nonhuman Primates and followed the ASP Code of Best Practices for Field Primatology.

## Conflicts of Interest

The authors declare no conflicts of interest.

## Data Availability

The data that support the findings of this study are available from the corresponding author upon reasonable request.
